# Yaws resurgence in Bankim, Cameroon: The relative effectiveness of different means of detection in rural communities

**DOI:** 10.1371/journal.pntd.0005557

**Published:** 2017-05-08

**Authors:** Alphonse Um Boock, Paschal Kum Awah, Ferdinand Mou, Mark Nichter

**Affiliations:** 1 FAIRMED Foundation, Yaoundé, Cameroon; 2 Paschal Kum Awah, Department of Anthropology, Faculty of Arts, Letters and Social Sciences, University of Yaoundé I, Cameroon; 3 School of Anthropology, University of Arizona, Tucson, Arizona, United States of America; University of Tennessee, UNITED STATES

## Abstract

**Background:**

Yaws is an infectious, debilitating and disfiguring disease of poverty that mainly affects children in rural communities in tropical areas. In Cameroon, mass-treatment campaigns carried out in the 1950s reduced yaws to such low levels that it was presumed the disease was eradicated. In 2010, an epidemiological study in Bankim Health District detected 29 cases of yaws. Five different means of detecting yaws in clinical and community settings were initiated in Bankim over the following five years.

**Methodology:**

This observational study reviews data on the number of cases of yaws identified by each of the five yaws detection approaches: 1) passive yaws detection at local clinics after staff attended Neglected Tropical Disease awareness workshops, 2) community-based case detection carried out in remote communities by hospital staff who relied on community health workers to identify cases, 3) yaws screening following mass Buruli Ulcer outreach programs being piloted in the district, 4) school-based screening programs conducted as stand-alone and follow-up activities to mass outreach events, and 5) house to house active surveillance activities conducted in thirty-eight communities. Implementation of each of the four community-based approaches was observed by a team of health social scientists tasked with assessing the strengths and limitations of each detection method.

**Findings:**

Eight hundred and fifteen cases of yaws were detected between 2012 and 2015. Only 7% were detected at local clinics. Small outreach programs and household surveys detected yaws in a broad spectrum of communities. The most successful means of yaws detection, accounting for over 70% of cases identified, were mass outreach programs and school based screenings in communities where yaws was detected.

**Conclusion:**

The five interventions for detecting yaws had a synergistic effect and proved to be valuable components of a yaws eradication program. Well planned, culturally sensitive mass outreach educational programs accompanied by school-based programs proved to be particularly effective in Bankim. Including yaws detection in a Buruli Ulcer outreach program constituted a win-win situation, as the demonstration effect of yaws treatment (rapid cure) increased confidence in early Buruli ulcer treatment. Mass outreach programs functioned as magnets for both diseases as well as other kinds of chronic wounds that future outreach programs need to address.

## Author summary

Yaws is an infectious and disfiguring disease of poverty primarily affecting children in rural communities in tropical areas. Yaws is easily treated by a single dose of antibiotics and is on the World Health Organization’s eradication list. Yaws was thought eradicated in the Cameroon in the 1950s following aggressive mass-treatment campaigns. In 2010, epidemiological research revealed a resurgence of the disease. This paper discusses the relative success of five different means of detecting yaws in rural areas of Bankim District between 2012 and 2015. While few cases of yaws were detected at local clinics during this time, many cases were detected in the community. The most successful means of detecting yaws were mass outreach programs designed to educate the public about neglected tropical diseases found in the region, and follow up school-based screening programs. These programs were supported by local chiefs and traditional healers and found to be the best way of increasing community awareness about yaws, motivating community health workers to participate in outreach, and fostering trust in the free medical treatment being provided.

## Introduction

Yaws is an infectious, debilitating, and disfiguring disease of poverty that mainly affects children and adolescents living in rural communities in tropical areas of Africa, the Pacific Islands, and Southeast Asia with high levels of rainfall. Caused by the spirochete bacteria *Treponema pallidum*, subspecies *pertenue* is closely related to syphilis and one of three endemic non-venereal treponemal diseases. The bacterium causes a chronic relapsing treponematosis characterized by highly contagious primary and secondary cutaneous lesions and non-contagious tertiary destructive lesions of the bones. Humans are the primary reservoir for yaws and transmission occurs through skin to skin contact with the exudate of lesions by those who have skin abrasions or cuts. Yaws is usually contracted in childhood (75% of cases occur before age 15) and infectious lesions are infrequent after the age of 30 [1, 2].

In the early stage of the disease, which may last from weeks to months, skin lesions are highly contagious and present differently by season with more open infectious lesions and *papillomatous frambesides* in the wet season and drier, scalier, maculopapular lesions in the dry season. Painful and itching lesions commonly appear on the upper and lower limbs, fingers, toes, soles of the feet, face, genital areas, and buttocks. The early stage is typically characterized by a single elevated primary lesion that develops a crust that is shed, followed by secondary lesions on other parts of the body. After 3–4 months lesions subside due to host immune response. The disease then becomes latent. In about 10% of untreated patients, the infection progresses to the tertiary stage characterized by destruction of tissue, bone, and cartilage resulting in disfigurement and disability.

Once widespread in the tropics, the incidence of yaws has been controlled though a combination of mass treatment with single dose of antibiotics (injectable benzathine benzylpenicillin) along with better hygiene and access to clean water. It has been estimated that yaws control efforts mounted by the World Health Organization (WHO) and United Nations International Children's Emergency Fund (UNICEF) resulted in up to a 95% reduction of the disease burden worldwide. Efforts are currently underway to eradicate the disease by 2020 following the Morges strategy, which calls for an initial mass treatment of endemic communities with Azithromycin in tablet form [2] followed by ongoing active community-based surveillance system and if required surveys every 3–6 months to detect and treat remaining cases of yaws and their contacts [3]

Yaws continues to be endemic in at least 13 countries globally, of which Cameroon is one [4]. Eradication will require better surveillance, health worker training, community outreach, and targeted mass drug treatment when and where necessary. In Cameroon, mass-treatment campaigns carried out in the 1950s reduced yaws to such low levels that it was presumed the disease was eradicated except among groups of pygmies living in the dense forest. In 2007 and 2008 outbreaks of yaws occurred among pygmy groups in Lomié health district. Cameroon’s National Neglected Tropical Disease (NTD) Control Program (covering Buruli ulcer (BU), leishmaniasis, yaws, and leprosy) working in conjunction with the NGO FAIRMED carried out an epidemiological survey in the district of Lomié in 2009. One hundred sixty-seven cases of yaws were detected in 35 small communities surveilled. Seventy five percent of cases were children under the age of 15 years with a majority between 9–11 years of age.

Yaws surveillance was not included in the routine disease surveillance system elsewhere in Cameroon and assumed to be a problem confined to the pigmy population. This changed when an epidemiological study of leprosy, yaws, and BU was carried out in Bankim district in 2010. The study entailed an intensive house-to-house survey conducted in late March to mid-April during which time 9,344 households were visited and 48,962 people examined. Twenty-nine confirmed cases of yaws were detected [5]. It became evident that either those afflicted with yaws were not coming to clinics for treatment or health staff were failing to recognize and treat the disease, confusing it perhaps for scabies.

As a follow up to the survey, three day NTD workshops were conducted by the National Disease Control Program in 2012 in Bankim and surrounding districts attended by hospital and clinic staff. The objective of the workshops was to better familiarize health workers with the signs of BU, leprosy, and yaws; encourage them to identify presumptive cases; and send swabs for laboratory confirmation. Disease control officers began visiting communities in 2012–2013 in an attempt to identify cases and inform community health workers (CHWs) about the disease. During these visits, CHWs were shown posters displaying the signs of yaws and BU and asked to identify suspected cases in their communities.

In 2013, an innovative community-based outreach program was launched in Bankim by the NGO FAIRMED working in conjunction with the government health service and the Stop Buruli Consortium. The three objectives of the outreach program were to raise awareness about BU, identify cases, especially early category one cases, and create collaborative relationships between clinic staff, CHWs, traditional healers, and local chiefs. Community health workers were mobilized and tasked with organizing mass community BU outreach events.

The culturally sensitive program that was introduced differed from previous outreach programs conducted in the Cameroon. In the past, information about BU was conveyed from health staff to the local population in a top down manner without community feedback elicited. The innovative program being piloted drew upon a year of formative research carried out by teams of social scientists in Bankim on patterns of health care seeking for BU and other chronic ulcers. The education program introduced went well beyond educating the public about the signs of BU. It employed a question and answer format that encouraged two-way dialogue between community members and health staff. Participants were shown before and after photographs of BU-related wounds depicting the healing process when appropriate treatment was followed. Time was allotted for testimonials by those cured of BU. Former patients attested to the quality of care they had received by clinic staff in what was described as a newly upgraded BU treatment program. Community members were also given explanations for all health staff actions including the collection of blood for disease confirmation. Following the educational program, screening by government health workers took place for those having lesions that were possible signs of BU. Although the focus of the outreach program was BU, many cases of yaws began to be detected. In communities where yaws was identified, teams returned and conducted school-based yaws screening and education programs.

This paper examines the relative utility of five approaches to yaws detection in rural settings of Cameroon:

1. Clinic-based passive case detection following awareness workshops
2. Community-based case detection through relatively small NTD related outreach activities
3. Community-based detection immediately following mass outreach events
4. School-based screening programs as stand-alone activities or as follow-up to mass outreach events in communities where yaws was identified
5. Community-based detection of yaws by labor intensive house to house active surveillance activities

We then present a brief overview of data collected on the distribution of yaws cases in the community and lessons learned about the best times to conduct yaws detection activities.

## Methods

**Fig 1:**
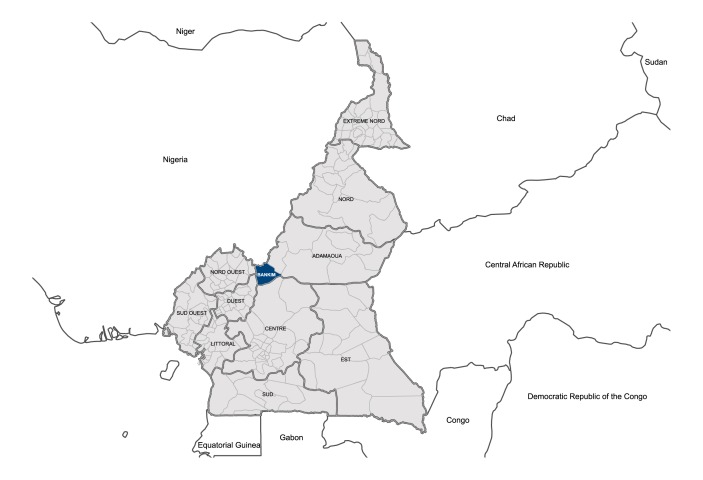
Map of Bankim district, Cameroon.

The study took place in Bankim district located in the northwest Adamawa region of Cameroon (Fig 1).

Bankim is situated in the Mape River Valley, where a hydro-electric dam was built more than twenty-five years ago. The Mape Dam splits the area into isolated islands and scattered communities. In the last two decades, increased irrigation has enabled rice cultivation. Inhabitants of the region also engage in growing maize, cassava, and peanuts as well as various forms of hunting and fishing. Much agriculture is done on plots of land some distance from the homes of community members during the months of January through May. Population movement and residence in the region is fluid and seasonal.

Bankim Health District is a challenging place to initiate a community outreach program due to both its rugged terrain and the wide variety of ethnic groups inhabiting the region. These groups speak a variety of languages and dialects in addition to French and Pidgin English. The district is served by a district hospital and 10 satellite clinics of which eight are government and two private and mission supported. All clinics report cases of yaws to Bankim hospital. Beginning in 2013 this hospital conducted rudimentary laboratory analysis for cases of yaws using Trepanoma Pallidum Hemagglutination Assay (TPHA), an inexpensive test indicative of, but not specific for, yaws.

Each of the five approaches for detecting yaws in Bankim health district described in this paper were implemented between January 2012 and December 2015. During this time a range of NTD related community outreach activities were ongoing involving local health staff and community health workers (CHWs) in different capacities. Health facility records of cases of yaws identified at Bankim hospital and local clinics were reviewed along with the records of yaws cases identified during small-scale NTD outreach activities documented by the disease control officer attached to Bankim Hospital.

Innovative community outreach programs for BU attracting large gatherings of community members took place from January 2013 through March 2015. During these programs trained health staff delivered culturally sensitive education programs developed and pretested by Cameroonian social scientists working with the Stop Buruli consortium. Outreach events generally attracted 400–600 participants and made use of image rich PowerPoint presentations conveying key messages about BU and its treatment. Meetings were organized by community health workers who also assisted in translating messages into local dialects. These events were attended by local chiefs, healers, and former patients who gave testimonials about the quality of care they had received.

Mass outreach events were generally held in the evening and lasted two to three hours. At the end of the event there was an opportunity for people to have their wounds screened for BU or to arrange for screening at a clinic. Local clinic staff and CHWs learned how to identify the signs of BU and yaws by observing trained hospital staff who used these events as teachable moments. CHWs were then encouraged to refer potential cases of yaws to clinics and screenings following future outreach events.

Patients suspected of having yaws were treated on the spot with free injections of benzathine penicillin. Blood samples from lesions were taken from a nonrandom sample of 120 patients by digital puncture or puncture at the heel and then transported to the laboratory of the Bankim hospital in 0.2 ml Eppendorf bottles with Ethylenediaminetetraacetic acid. Two tests were carried out by an experienced laboratory technician: rapid plasma regain (RPR) and TPHA. Both are routinely used and reactive in the screening of yaws and syphilis.

In communities where cases of yaws were detected during BU outreach events, health staff followed up with school-based yaws education and detection programs. Data on the age and gender of yaws patients were recorded along with information on school attendance and the clustering of cases in particular households and communities. In 2015, there was a gap in funding for mass BU outreach events. During this time another source of funds became available for two intensive house to house NTD surveys conducted in the months of August and November. One thousand eight hundred and eighty-nine (1,889) households residing in thirty eight communities were visited by health staff.

### Ethical considerations

The National Ethics Committee for Health Research overseen by the Cameroon Ministry of Public Health Cameroon approved this study. All study participants voluntarily opted into the study through documented informed consent. In cases where children were interviewed or their blood was drawn for testing, consent forms were secured from parents after being informed why a test was being administered.

## Results

Eight hundred and fifteen (815) cases of suspected yaws were detected between 2012 and 2015 in Bankim district. Blood samples from 120-suspected cases were sent to Bankim Health District Laboratory for testing, of which 100 cases were < 16 years of age, 16 were 16–30 years of age, and four over > 30 years of age. The RPR confirmation rate was 85% and the TPHA confirmation rate 77%. It is possible that some of the remaining 23% of cases included people treated for yaws or syphilis in the past. All 815 cases were followed up four weeks after antibiotic treatment was administered. Complete recovery was observed in 89% of cases with the remaining 11% of cases found symptom free three weeks later.

Tables 1 and 2 summarize how the 815 yaws cases were detected. As a means of assessing the cumulative effect of the four community outreach activities, it may be noted that no yaws cases were recorded in Bankim district in the five years prior to 2012, when outreach activities were initiated. Furthermore, between 2012 and 2015 only four cases of yaws were reported at clinics in the neighboring district of Malentouen, although health staff in this district had attended a three-day NTD workshop in 2012 alerting them to the presence of yaws in the region. In Malentouten district, community based outreach activities had yet been introduced.

**Table 1:** Detection method and number of yaws cases detected yearly from 2012–2015.

Year	Detection methods								
	Passive detection at clinics	NTD community outreach Programs		Mass BU program followed by school screening		School-based program alone		House-to-house search	
		Communities visited	Cases of yaws found	Mass BU program activities	Cases of yaws found	Schools visited	Cases of yaws found	No of house-holds visited	Cases of yaws found
2012	8	4	13	0	0	5	40	0	0
2013	12	2	18	22	97	0	0	0	0
2014	18	10	33	18	161	4	27	0	0
2015	22	0	0	4	70	19	186	1889	110

**Table 2:** Yearly distribution of yaws cases by detection methods.

Year	Detection methods									
	Passive detection at clinics		NTD community outreach Programs		Mass BU program followed by school screening		School-based program alone		House-to-house search (no of households visited	
	Freq.	Perc.	Freq.	Perc.	Freq.	Perc.	Freq.	Perc.	Freq.	Perc.
2012	8	13%	13	21.3%	0	0	40	65.6%	0	0
2013	12	9.4%	18	14.1%	97	76.3%	0	0	0	0
2014	18	7.5%	33	13.8%	161	67.4%	27	11.29%	0	0
2015	22	6%	0	0	70	18.04%	186	48%	110	28.3%
Total	60	7.36%	64	7.85%	328	40.25%	253	31.04%	110	13.50%

Five observations may be highlighted. First, even after yaws awareness training for health staff working in clinics and three years of outreach activities where health staff encouraged community members with yaws-like symptoms to visit clinics, only 7% of all yaws cases were detected at clinics. This suggests that community members with yaws-like symptoms are not commonly visiting clinics for treatment (see Agana-Nsiire [6] for a similar finding in Ghana). Ethnographic research confirmed this observation. The symptoms of yaws (itching and moderate levels of pain) are not seen to be serious enough to warrant seeking care at a clinic, especially if a clinic is distant. The fact that the symptoms of yaws eventually subside (as the disease becomes latent) led some community members to conclude that the disease was self-limiting, recurrent, or seasonal. Despite a rising level of awareness within the local population about yaws and the effectiveness of drug therapy resulting from outreach programs, most community members afflicted with yaws-like symptoms preferred to wait for outreach screening events rather than travel to clinics. This is evidenced by clinic data that documents only a small increase in yaws cases seen at clinics in Bankim during the four-year period.

Second, NTD outreach activities in remote communities identified yaws cases largely based on the mobilization efforts of CHWs. In 2012 and 2013 health staff visited six small to moderate sized (< 100 households) remote communities in December and January. Thirty-one cases of yaws were identified and treated. In December–January 2014, the Stop Buruli team visited another 10 remote communities (of similar size) searching for both BU and yaws cases. CHWs exposed to basic information about the two diseases were asked to identify possible cases in their community. Together with health staff, CHWs detected sixty cases of yaws. In total 11% of all cases of yaws were identified through this outreach approach, yielding a mean of 5.5 cases per outreach activity.

Third, a big spike in yaws detection occurred with the initiation of mass BU outreach program events followed by school screenings in communities where yaws was detected. These programs were held in mid-November through January, months when a majority of the local population reside in their homes and are not engaged in agricultural activities some distance away. Programs were conducted in moderate to large sized communities with schools. They were held in the early evening, attended by community leaders, and seen by community members as a major event. Light entertainment preceded the education program.

At first, the outreach team only focused on BU and did not pay much attention to other kinds of skin lesions. However, after a number of yaws cases were detected among children in 2013, the decision was made to be more proactive and screen for yaws. Between 2013 and 2015, 44 screenings were held after mass BU outreach events. When cases of yaws were identified in a community, school screenings were arranged and carried out. Three hundred and twenty-eight cases of yaws were detected with a mean yield of 9.4 case per mass event/school screening activity set. Notably, the case yield increased over time as people came to see health staff as accessible and free medication available for both yaws and BU. In 2013, 4.4 cases of yaws per activity set were detected. In 2014 the case yield was 8.9, and in 2015 17.5 cases.

A fourth observation focuses attention on the importance of school based yaws detection programs. Twenty-seven percent of all yaws cases detected between 2012 and 2015 were identified during stand-alone school screenings in communities where no mass BU outreach event had been held. In 2012, school programs held in five moderate sized communities yielded 8 cases of yaws per activity. By 2015, stand-alone school screening events yielded 9.8 cases per screening. Social scientists found that large BU outreach events supported by community leaders in nearby communities helped legitimize school- based screening programs. The parents of children had a much better idea of why screenings in schools were being held and had confidence in the medication offered given the circulation of stories of successful yaws treatment.

In addition to detecting cases at schools, school-based programs taught students how to identify the signs of yaws. The social science team investigated whether identifying children with yaws in the school would be stigmatizing. Observations and interviews with children did not find this to be the case. Those conducting the education program made it clear that yaws was easily treated with just one injection and the demonstration effect of classmates recovering rapidly from symptoms made the program popular. By 2014, students were asked to examine each other for “tell-tale signs” of yaws and encouraged to identify possible cases of yaws in children either too young to attend school or who stopped going to school as a result of painful or unsightly lesions. In short, schoolchildren were enlisted to assist in community based identification of yaws among their peers and those children they helped care for at home.

A fifth observation entails the effectiveness of house-to-house surveys as a strategy for achieving yaws eradication in rural areas of Cameroon. As the result of an unfortunate break in funding in 2015, Stop Buruli mass outreach activities were suspended. To keep up the momentum of NTD activities, FAIRMED in conjunction with Cameroon’s government NTD program conducted school based screenings as well as an intensive house-to-house NTD survey. The two phase survey was carried out during late August and mid-November. In all, 1,889 houses in 38 communities were canvassed and 110 cases of yaws detected, 13% of total yaws cases identified between 2012 and 2015. Two points may be made. First, the number of yaws cases in the 2015 survey far exceeded the number of cases detected in 2010, when a much larger survey (N = 9,344) was carried out in the month of March. March is a busy month for agriculturalists and many people are working in fields far from their community. In 2015, yaws was detected in 6% of all households while in the 2010 survey cases were found in only .3% of households visited. A second point was that the program required a significant investment of health staff. In 2015, the participation of seven hospital staff members was required for 10 days of arduous surveillance activities. This constituted a significant opportunity cost for a busy district hospital like Bankim, which serves a population of about 100,000 inhabitants. Table 3 summarizes the relative advantages, limitations and logistical challenges of each type of yaws detection activity observed.

**Table 3:** Advantages and limitations of five ways of detecting yaws.

Means of detecting yaws	What the approach adds	Limitations and logistics needed	Comments
Passive detection at clinic following awareness workshops and continuing education	Increased clinic staff awareness of yaws; building confidence in diagnosing and treating yaws	Few of those afflicted with yaws appear to visit clinics for this ailment	It would be productive to trace yaws cases back to their communities and implement recommended eradication protocols
NTD outreach by clinic staff to remote communities	Raising of CHW awareness of yaws; on site treatment; necessary to reach remote communities	Staff can only reach a small percentage of communities; high cost in staff time and resources; significant opportunity costs	• Relies on active CHW involvement in identifying potential cases • Could be combined/integrated with other public health activities like vaccination campaigns–if they are well received by the community
Innovative mass outreach events for NTDs like BU	Culturally sensitive education using question: answer format driven by formative research responds to local perceptions and concerns; support by local leaders, healers; raises status of CHW and increases their motivation to be proactive; translation of information across languages when necessary; on site screening and treatment	• Requires resources and coordination between CHW and health staff; support of local leaders • Only offering free treatment for select diseases and not all chronic skin diseases difficult for community members to understand	• Serves as a magnet drawing large crowds and those afflicted with a range of skin diseases; legitimizes school based screening and treatment programs • Combine with: school based follow up screening programs for yaws cases; wound care education programs
School based programs	Reaches school children as group at risk; school children learn to recognize signs of yaws; children attending school identify other children with signs of yaws not attending school	Cooperation of teachers and trust of parents is necessary in order for children to be treated on site	Need to be sure stigmatization does not occur when yaws cases are identified in school; can be combined/integrated with other school health activities such as basic wound and skin care practices
House to house surveys	Blanket coverage if residents are at home	High cost in staff time and resources; significant, opportunity costs; needs to be carried out in season when maximum number of people are in residence	• Might be called for in high yaws prevalence communities if mass drug distribution is required • May be combined/integrated with other public health activities like vaccination campaigns

## Observations of the distribution of yaws in Bankim

**Fig 2:**
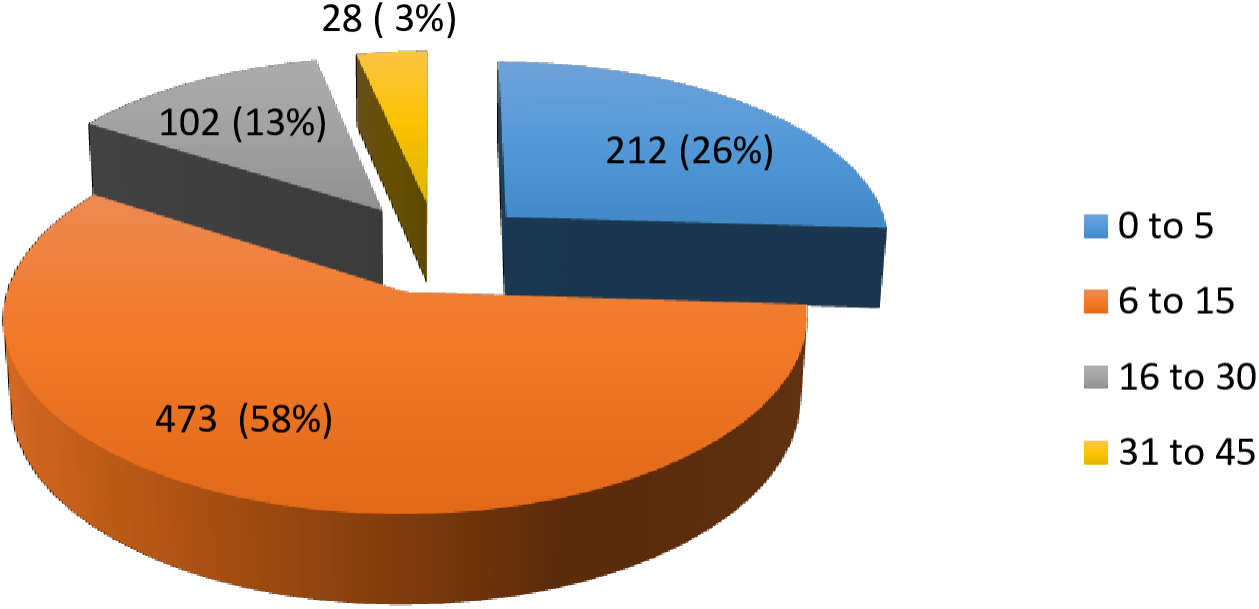
Distribution of yaws cases by age.

Data was collected on the distribution of yaws cases by age, gender, ethnic group, and household occupation as well as season. An analysis of cases of yaws by age conforms to a well-described epidemiological pattern (Fig 2). Eighty-four percent (84%) of yaws cases were under 15 years of age with 26% of children being under the age of 5 years. The large number of young children suffering from yaws suggests that school-based programs alone are insufficient to reach a significant percentage of high-risk children.

The gender distribution of children detected with yaws is presented in Table 4. We found a greater number of male cases in all age categories over the course of the four years of the study.

**Table 4:** Gender distribution of yaws cases by age groups in children under 16 years of age.

	0 to 5 years		6 to 15 years		Total	
	# of cases	%	# of cases	%	# of cases	%
Male	133	57%	303	67%	436	64%
Female	101	43%	148	33%	249	36%
**Total**	**234**	**100%**	**451**	**100%**	**685**	**100%**

In Bankim, as in much of West Africa, sibling care is common. Sixty-six percent (66%) of all children symptomatic for yaws are of school-going age. They not only have frequent skin to skin contact with classmates in school, but younger siblings. Observations of sibling childcare by social scientists documented that while school-going females more commonly care for younger siblings, school aged males do so as well. Breaking the chain of transmission required teaching school children how to recognize the signs of yaws in their siblings.

The most common types of yaws lesions detected between 2012 and 2015 are presented in Table 5. Notably, the most common lesions are also the most contagious (ulcers—69% and papilloma—19%).

**Table 5:** Clinical manifestations of yaws.

	2012		2013		2014		2015		TOTAL	
Lesions observed	N	%	N	%	N	%	N	%	N	%
Papilloma	21	24	23	23	82	21	141	17	267	19
Ulcer	66	75	77	77	243	63	570	70	956	69
Maculopapular rash	1	1	0	0	29	7	47	6	77	6
Dactilitis	0	0	0	0	11	3	16	2	27	2
Palmar/plantar hyperkeratosis	0	0	0	0	23	6	41	5	64	4
Total lesions observed	88	100	100	100	388	100	815	100	1391	100

We examined the distribution of yaws cases by locale as well as ethnic and occupational group. Data on the six health areas that comprise Bankim district revealed that yaws cases were widely distributed with hot spots in both towns and villages. The maximum number of cases was detected in locales proximate to the Mbam River and Mape dam.

Analysis of data on yaws cases by ethnic groups likewise revealed broad distribution of cases across the six largest ethnic groups in the district as distinct from clustering in any particular group. One finds both single and mixed ethnic group settlements in Bankim. The largest ethnic groups were the groups with the most exposure to outreach programs and the most cases of yaws detected. Analysis by occupation found broad distribution across communities that rely on both agriculture and fishing.

We next looked at yaws cases detected by month. The majority of cases were identified during outreach screening activities when community members were more likely to be at home. Although carried out throughout the year, outreach activities were easier to conduct in some seasons due to climate, transportation, agricultural cycles, school registration, and ritual activities in the region. Peak months of yaws detection in the community were August–September and November–December. Fewer cases were detected from January to June.

## Discussion

Over the last decade, there have been repeated calls for integrated NTD programs [7, 8, 9] including NTDs that cause skin lesions [10, 11]. To date, most examples of integration have entailed the integration of preventive chemotherapy programs [12, 13] including those that have used schools as sites for community-based NTD control activities [14, 15, 16]. In Bankim district NTD integration evolved in response to a community based outreach program for BU. Few cases of yaws were diagnosed in the district until a community based outreach program for BU was initiated attracting large gatherings of community members. These gatherings proved to be magnets for community members with chronic lesions and wounds. While yaws cases was not an initial focus of wound screening, this changed when many cases of yaws were detected, much to the surprise of health staff. Offering free treatment for yaws proved to be a win-win scenario for both BU and yaws outreach. The effectiveness of yaws treatment increased confidence in medications offered to treat cases of BU.

The five interventions of yaws detection profiled in this study were found to have a synergistic effect and constitute valuable approaches to eradicating yaws. The most time and cost effective means of outreach in Bankim were well-planned mass education programs followed up by school-based programs. Piggy backing yaws eradication onto BU outreach programs made sense in Bankim because mass events are given far more importance by community members than short outreach activities conducted solely by health staff. These events are attended by chiefs and influential traditional healers who offer support, increase program credibility, and motivate CHWs to be more proactive. They also boost community trust in school based NTD activities that other studies have found do better when accompanied by broader based community outreach [17].

In Bankim, outreach activities carried out in schools not only served as opportunities for identifying cases of yaws among the segment of the population most likely to suffer from yaws, but educated children to detect yaws in the future. Children not only brought health messages home to parents, they were trained to detect yaws among children too young to attend school. Given that school-aged children of both genders commonly engage in sibling care, they are both a good detection resource and an important link in the chain of yaws transmission. Teachers were also found to be a good source of knowledge about children who appear to have dropped out of school due to skin diseases.

### Limitations

In this study, we ascertained the relative effectiveness of five methods of detecting yaws from a review of records of patients treated for the disease in different clinic and community contexts. One of the lessons learned is that single NTD disease focused outreach programs, like the mass BU events described in this paper, attracts community members with a wide range of chronic skin diseases like yaws. We initially identified yaws cases serendipitously. Over time we felt the need to be more proactive in identifying yaws cases, as we deemed it unethical not to do so. A limitation of the project was that we did not add yaws messages to the mass BU outreach events given that the novel BU education approach piloted was being evaluated. The addition of yaws messages might have constituted a confounding variable negatively impacting BU message evaluation. Furthermore, we had not conducted formative research on yaws necessary for the design of culturally appropriate messages. Basic yaws recognition was included in school-based programs. In the near future, we will integrate yaws messages into all community and school based NTD education programs once such messages are pretested.

### Conclusion

WHO recommendations for eradicating yaws include mass treatment with oral azithromycin, the use of recently developed rapid diagnostic tests, and three to six month follow up in endemic communities. Cameroon is in the process of adopting these measures as soon as resources and manpower become available. In remote locations like Bankim, yaws eradication will prove challenging due to poor transportation, population movements, and ethnic diversity requiring that education to be delivered in multiple languages. The five kinds of interventions described in this paper will need to be coupled with mass treatment strategies in order to achieve the level of community outreach needed for eradication.

Yaws eradication may also require a more comprehensive approach to neglected tropical skin diseases and wound care. During Bankim outreach activities, health staff encountered many cases of chronic wounds that were either neglected or being treated inappropriately, leading to complications. Only focusing on BU and yaws and neglecting other wounds and lesions sends a message to the community that some wounds and skin diseases matter more than others, and that treating specific diseases matters more than providing care to people suffering from debilitating skin conditions. Better diagnostic testing will only add to this perception as more lesions, that to community members look similar to yaws or BU, are ruled out for free treatment [18, 19, 20].

Outreach programs in community and school settings combining NTD disease surveillance with wound care education, and when appropriate free treatment, would help address this problem. Such programs would serve a broader primary health care agenda [21] and minimize the kinds of rumors that have undermined other NTD programs in Africa [22, 23, 24] including BU [25]. In West Africa, local perceptions of illness and the agenda of those conducting public health programs matter. In order to be sustainable, NTD programs will need to build community trust. Much of the success of the Bankim program can be attributed to concerted efforts to respect and involve all community stakeholders in NTD activities.

## Supporting information

S1 Checklist STROBE statement—Checklist of items that should be included in reports of observational studies. (DOC)
