# Supplementary material for: Yaws resurgence in Bankim, Cameroon: The relative effectiveness of different means of detection in rural communities
Source: PLoS Negl Trop Dis. 2017 May 8;11(5):e0005557. doi: 10.1371/journal.pntd.0005557 (PMC5436870; doi:10.1371/journal.pntd.0005557)
Supplement: S1 Checklist — (DOC) [file pntd.0005557.s001.doc]

STROBE Statement—checklist of items that should be included in reports of observational studies

|  | Item No | Recommendation |
| --- | --- | --- |
| **Title and abstract**  **In abstract- after conferring with a Plos editors (Christain and Pam Small)**  **study described as observational and not cross -sectional** | 1 | (*a*) Indicate the study’s design with a commonly used term in the title or the abstract |
|  |  | (*b*) Provide in the abstract an informative and balanced summary of what was done and what was found –Included and cut down in size |
| Introduction | | |
| Background/rationale _Included …  re-emergence of yaws , but disease not detected at clinics | 2 | Explain the scientific background and rationale for the investigation being reported In background |
| Objectives  To look at relative effectiveness of different  ways of detecting yaws in the community –clearly stated | 3 | State specific objectives, including any prespecified hypotheses No Hypothesis |
| Methods | | |
| Study design  Included | 4 | Present key elements of study design early in the paper |
| Setting  Described in detail | 5 | Describe the setting, locations, and relevant dates, including periods of recruitment, exposure, follow-up, and data collection |
| Participants  These categories do not apply to this study | 6 | (*a*) *Cohort study*—Give the eligibility criteria, and the sources and methods of selection of participants. Describe methods of follow-up  *Case-control study*—Give the eligibility criteria, and the sources and methods of case ascertainment and control selection. Give the rationale for the choice of cases and controls  *Cross-sectional study*—Give the eligibility criteria, and the sources and methods of selection of participants |
|  |  | (*b*) *Cohort study*—For matched studies, give matching criteria and number of exposed and unexposed  *Case-control study*—For matched studies, give matching criteria and the number of controls per case |
| Variables  Outcomes described : cases of suspected yaws detected by method of detection  Yaws cases confirmed by specified tests | 7 | Clearly define all outcomes, exposures, predictors, potential confounders, and effect modifiers. Give diagnostic criteria, if applicable |
| Data sources/ measurement  Sources of data described  Clinic and outreach program treatment records, lab tests, and field observations by social scientists | 8* | For each variable of interest, give sources of data and details of methods of assessment (measurement). Describe comparability of assessment methods if there is more than one group |
| Bias  No bias detected -- yaws case records | 9 | Describe any efforts to address potential sources of bias |
| Study size  Does not apply, all cases of yaws in records during specified time period included | 10 | Explain how the study size was arrived at |
| Quantitative variables  Simple count of cases of suspected yaws | 11 | Explain how quantitative variables were handled in the analyses. If applicable, describe which groupings were chosen and why |
| Statistical methods  Only percentages used | 12 | (*a*) Describe all statistical methods, including those used to control for confounding |
|  |  | (*b*) Describe any methods used to examine subgroups and interactions |
|  |  | (*c*) Explain how missing data were addressed |
|  |  | (*d*) *Cohort study*—If applicable, explain how loss to follow-up was addressed  *Case-control study*—If applicable, explain how matching of cases and controls was addressed  *Cross-sectional study*—If applicable, describe analytical methods taking account of sampling strategy |
|  |  | (*e*) Describe any sensitivity analyses |

Continued on next page

| Results | | |
| --- | --- | --- |
| Participants  Case records and observations of out reach activities involving large groups | 13* | (a) Report numbers of individuals at each stage of study—eg numbers potentially eligible, examined for eligibility, confirmed eligible, included in the study, completing follow-up, and analysed |
|  |  | (b) Give reasons for non-participation at each stage |
|  |  | (c) Consider use of a flow diagram |
| Descriptive data  Age and gender of yaws cases detected are described  in a table | 14* | (a) Give characteristics of study participants (eg demographic, clinical, social) and information on exposures and potential confounders |
|  |  | (b) Indicate number of participants with missing data for each variable of interest |
|  |  | (c) *Cohort study*—Summarise follow-up time (eg, average and total amount) |
| Outcome data  Included in tables 1-2 | 15* | *Cohort study*—Report numbers of outcome events or summary measures over time |
|  |  | *Case-control study—*Report numbers in each exposure category, or summary measures of exposure |
|  |  | *Cross-sectional study—*Report numbers of outcome events or summary measures |
| Main results : included  Queries about statistics are not relevant | 16 | (*a*) Give unadjusted estimates and, if applicable, confounder-adjusted estimates and their precision (eg, 95% confidence interval). Make clear which confounders were adjusted for and why they were included |
|  |  | (*b*) Report category boundaries when continuous variables were categorized |
|  |  | (*c*) If relevant, consider translating estimates of relative risk into absolute risk for a meaningful time period |
| Other analyses  Confirmation of subsample of yaws cases by laboratory procedures reported | 17 | Report other analyses done—eg analyses of subgroups and interactions, and sensitivity analyses |
| Discussion | | |
| Key results clearly stated | 18 | Summarise key results with reference to study objectives |
| Limitations – section provided –limitation section discussed with editor Pam Small | 19 | Discuss limitations of the study, taking into account sources of potential bias or imprecision. Discuss both direction and magnitude of any potential bias |
| Interpretation offered in discussion and conclusion | 20 | Give a cautious overall interpretation of results considering objectives, limitations, multiplicity of analyses, results from similar studies, and other relevant evidence |
| Generalisability –  In discussion re. the merits of the 5 methods of detecting yaws was and was deemed most robust | 21 | Discuss the generalisability (external validity) of the study results |
| Other information | | |
| Funding Provided | 22 | Give the source of funding and the role of the funders for the present study and, if applicable, for the original study on which the present article is based |

*Give information separately for cases and controls in case-control studies and, if applicable, for exposed and unexposed groups in cohort and cross-sectional studies.

**Note:** An Explanation and Elaboration article discusses each checklist item and gives methodological background and published examples of transparent reporting. The STROBE checklist is best used in conjunction with this article (freely available on the Web sites of PLoS Medicine at http://www.plosmedicine.org/, Annals of Internal Medicine at http://www.annals.org/, and Epidemiology at http://www.epidem.com/). Information on the STROBE Initiative is available at www.strobe-statement.org.
